# A groupwise multiresolution network for DCE-MRI image registration

**DOI:** 10.1038/s41598-025-94275-9

**Published:** 2025-03-22

**Authors:** Anika Strittmatter, Meike Weis, Frank G. Zöllner

**Affiliations:** 1 Computer Assisted Clinical Medicine, Medical Faculty Mannheim, Heidelberg University, Theodor-Kutzer-Ufer 1-3, 68167 Mannheim, Germany; 2 Mannheim Institute for Intelligent Systems in Medicine, Medical Faculty Mannheim, Heidelberg University, Theodor-Kutzer-Ufer 1-3, 68167 Mannheim, Germany; 3 Department of Clinical Radiology and Nuclear Medicine, University Medical Center Mannheim, Medical Faculty Mannheim, Heidelberg University, Theodor-Kutzer-Ufer 1-3, 68167 Mannheim, Germany

**Keywords:** Deep learning, Image registration, Machine learning, Medical images, Groupwise, Multiresolution, Computational science, Computer science, Magnetic resonance imaging, Health care, Mathematics and computing

## Abstract

In four-dimensional time series such as dynamic contrast-enhanced (DCE) MRI, motion between individual time steps due to the patient’s breathing or movement leads to incorrect image analysis, e.g., when calculating perfusion. Image registration of the volumes of the individual time steps is necessary to improve the accuracy of the subsequent image analysis. Both groupwise and multiresolution registration methods have shown great potential for medical image registration. To combine the advantages of groupwise and multiresolution registration, we proposed a groupwise multiresolution network for deformable medical image registration. We applied our proposed method to the registration of DCE-MR images for the assessment of lung perfusion in patients with congenital diaphragmatic hernia. The networks were trained unsupervised with Mutual Information and Gradient L2 loss. We compared the groupwise networks with a pairwise deformable registration network and a published groupwise network as benchmarks and the classical registration method SimpleElastix as baseline using four-dimensional DCE-MR scans of patients after congenital diaphragmatic hernia repair. Experimental results showed that our groupwise network yields results with high spatial alignment (SSIM up to 0.953 ± 0.025 or 0.936 ± 0.028 respectively), medically plausible transformation with low image folding (|J| ≤ 0: 0.0 ± 0.0%), and a low registration time of less than 10 seconds for a four-dimensional DCE-MR scan with 50 time steps. Furthermore, our results demonstrate that image registration with the proposed groupwise network enhances the accuracy of medical image analysis by leading to more homogeneous perfusion maps.

## Introduction

In image registration, corresponding points or structures of two or more images are spatially aligned. For instance, in co-registration, multiple images of different acquisitions or modalities can be spatially aligned, which enables the fusion of the images and their (complementary) information^1^, e.g. the fusion of computer tomography (CT) and magnetic resonance (MR) scans for multimodal minimal-invasive image-guided interventions^2^. In addition, image registration can be used to correct movement between different scans of the same acquisition. For instance, in four-dimensional time series such as dynamic contrast-enhanced (DCE) MRI, the motion between individual time steps due to the patient’s breathing or movement can be corrected. Therefore image registration is an important step in medical image analysis and helps to improve various applications.

One medical application that benefits from image registration is the assessment of lung perfusion in patients with congenital diaphragmatic hernia (CDH). Briefly, CDH is a birth defect in which the diaphragm did not close completely during the foetal development^3^. The foetal abdominal organs are partially located in the thoracic cavity, which then leads to impaired lung development and can also result in cardiovascular malformations. Prenatal diagnosis of diaphragmatic hernia plays a crucial role and allows early planning for prenatal interventions^4^. In addition to prenatal ultrasound imaging, foetal MRI is also used to examine the pulmonary function as well as the lung anatomy. Dynamic contrast-enhanced (DCE) MRI can be used to determine quantitative parameters of lung perfusion^5^. This involves acquiring a time series of three-dimensional MR scans to monitor the distribution of the administered contrast agent. The four-dimensional data can then be used to calculate perfusion maps, e.g. mean transit time, pulmonary blood volume or pulmonary blood flow^6^.

In patients with CDH, DCE-MRI is performed in free-breathing. Due to the patient’s respiration between the individual time step scans, the patient is at a different point in the respiratory cycle in each time step scan. This results in a varying deformation of the organs, such as the lungs, heart and liver in each time step. The varying deformation of the corresponding structures in the individual time steps leads to an inaccuracy in the resulting perfusion map. By spatially aligning the corresponding structures before calculating the perfusion maps, the accuracy of the resulting perfusion map can be improved.

Some published methods for the registration of DCE-MR images like Ruthotto et al.^7^ and Cai et al.^8^ apply pairwise registration, whereby one volume is defined as a reference/fixed image and the other volumes are defined as moving images. During registration, a transformation is calculated for each moving image in order to spatially align the moving images with the fixed image. The moving images are then transformed with the calculated transformations, resulting in the so-called moved images. Careful selection of the fixed image is crucial as, e.g., choosing a volume with significant motion (e.g., sudden patient movement) can lead to excessive deformations in the registration process. Even if the other volumes have only minor deformations relative to each other, an unrepresentative fixed image can result in unnecessarily large deformations across the entire four-dimensional scan. Instead, for four-dimensional images like DCE-MR scans, groupwise registration can be applied, where more than two images are spatially aligned^9–11^. Setting a fixed image is usually omitted, thus avoiding the selection bias of a fixed image^12^. Instead, a template image is calculated from the volumes to be registered, e.g. the average intensity image. During registration, a transformation is calculated for all volumes to align them with the template image.

Published methods for the registration of DCE-MR images only use a single resolution. However, particularly in the field of medical image registration, the deformation cannot usually be corrected in a single step with a single resolution^13^. Instead, medical image registration often utilises methods that apply multiple resolutions, such as the classical registration method SimpleElastix^14^, or deep learning-based registration methods^15–21^.

However, to the best of our knowledge, no registration method has yet been published that performs groupwise and multiresolution registration of DCE-MR images. Therefore, to achieve accurate four-dimensional image registration, specifically on DCE-MR scans, we propose a novel groupwise deformable registration framework that utilises multiple resolutions, thus combining the advantages of groupwise and multiresolution registration. For our proposed groupwise registration network, the template image is calculated by averaging the intensities of all volumes to be registered. The groupwise registration network can be implemented with an arbitrary group size. We performed extensive experiments and evaluated the groupwise registration network with two different group sizes: groups of 5 and groups of 10 three-dimensional volumes from the four-dimensional DCE-MR dataset. We analyse the effect of groupwise registration by comparing it with pairwise registration using the same network architecture. Moreover, we compared the performance of the proposed groupwise registration network with a pairwise and groupwise registration using the classical registration method SimpleElastix as a baseline and the published groupwise network GroupRegNet^22^ to show the superiority of the proposed registration network.

## Methods

### Dataset

The dataset of this retrospective study comprises 30 four-dimensional DCE-MR scans of 2-year-old patients after CDH repair following a standardized protocol as described by Weidner et al.^5,23^. Briefly, each patient underwent a DCE-MR scan using a three-dimensional TWIST sequence for DCE-MRI lung perfusion imaging at a 3T scanner. The three-dimensional scans were obtained using a matrix of [126-162] x 192 voxels with 52-56 slices reconstructed to an isotropic voxel size of 1.97 mm$^3$. In total, 50 time steps, i.e. volumes, were acquired with a temporal resolution of 1.5 s.

Contrast agent (Dotarem, Guerbet, France) was injected at a flow rate of 1 mL/s after the fifth time step at a dose of approximately 1 mL (0.05 mmol/kg body weight) of the contrast agent mixed with an equal volume of sodium chloride. The contrast agent injection was immediately succeeded by a 10 mL bolus of sodium chloride^24^. All acquisitions were acquired in free breathing, the patients received sedation. This study was performed in accordance with ethical guidelines and regulations. This study and the experimental protocol were approved by the local research ethics committee Ethik-Kommission II of Heidelberg University. Informed parental consent was obtained for all subjects who participated in this study.

For the first three-dimensional volume (first time step), lung segmentations were created semi-automatically using a segmentation tool^24^ implemented in the DICOM Workstation OsiriX^25^.

### Network architectures

**Fig. 1:**
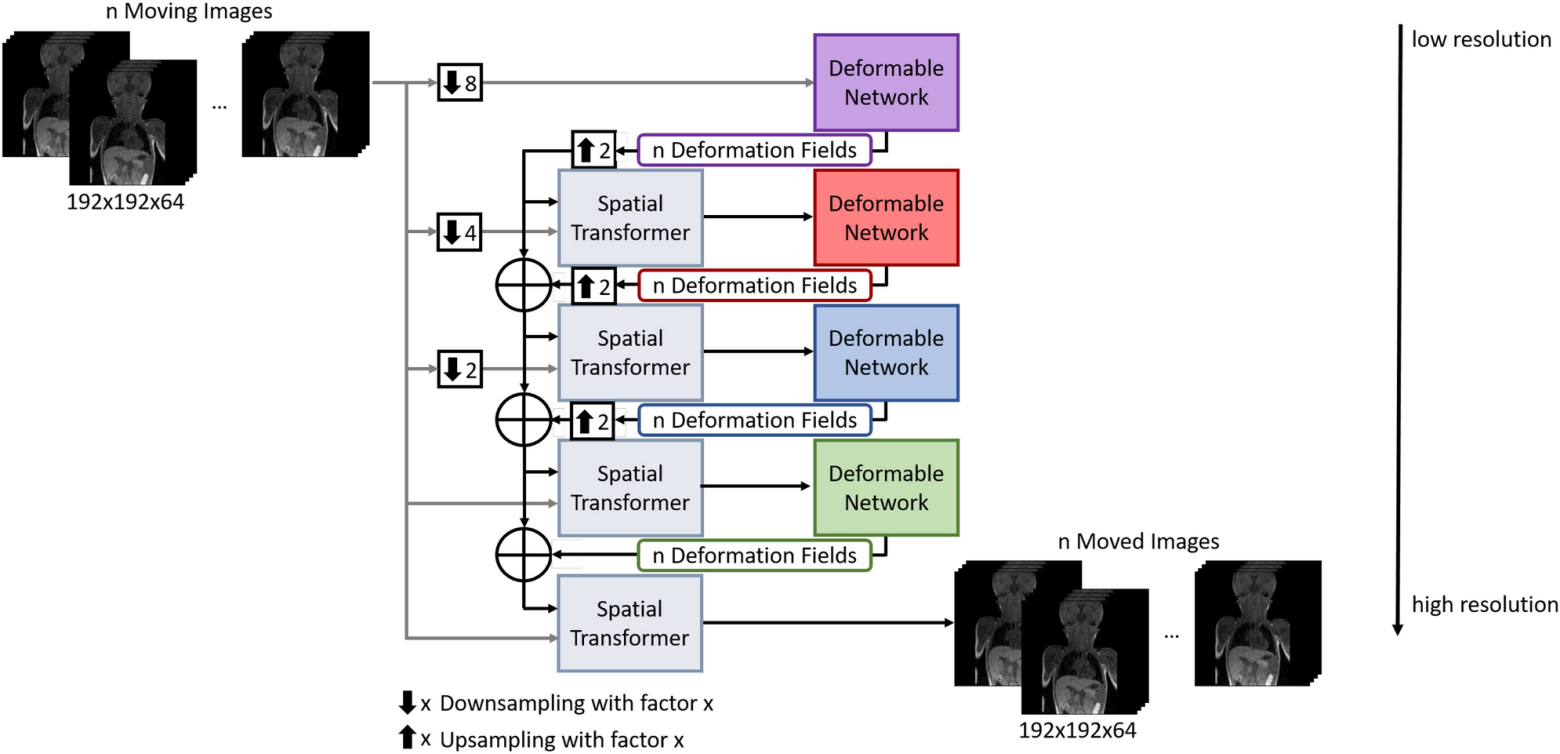
Architecture of the groupwise multistep network. The proposed method follows a coarse-to-fine approach: the coarse stages are located at the top of the figure, the subsequent stages with increasingly higher resolution are located further down in the figure. As subnetworks, we adapted VoxelMorph-2^21^. No normalization is used between layers.

Figure 1 shows the architecture of the groupwise deformable registration network. The group size n, i.e. the number of volumes to be registered, can be chosen arbitrarily. The volumes to be registered are concatenated and form the input of the groupwise registration network.

As underlying network architecture, we utilised a multiresolution network^21^, as this demonstrated good performance for various multimodal medical imaging datasets and applications. Four subnetworks are used, which process the volumes in increasingly higher resolution. Each subnetwork calculates n deformation fields, i.e. one deformation field per volume to be registered. Subsequent subnetworks then process the previously transformed volume using the deformation fields of the preceding subnetworks. The aim is to first reduce coarser deformations in the first subnetworks and then to correct increasingly finer deformations in the subsequent subnetworks with increasingly higher resolution. The calculated tranformation is applied to the volumes using a spatial transformer^26^, resulting in the moved volumes.

### Benchmark networks

As a benchmark network, we applied a pairwise deformable registration^21^ with the same network architecture as the groupwise registration. Here, two volumes, the fixed (time step 1) and the moving image (remaining time steps), are spatially aligned. A transformation is calculated, which is then applied to the moving image using the spatial transformer. Furthermore, we utilised GroupRegNet^22^ as an additional benchmark network.

### Training setting for the neural networks

We trained the networks unsupervised using Mutual Information (MI) and Gradient $L_2$ as loss functions.

Our proposed network applies groupwise registration. For this, a template image T is used, which is calculated by averaging the intensities of all volumes to be registered. In groupwise registration, the Mutual Information is calculated between each moved image M and the template image T:

1 $$\begin{aligned} L_{MI}(T, M) = I (T; M) = H(T) + H(M) - H(T, M), \end{aligned}$$

where H(T) denotes the Shannon entropy of the template image T and H(M) denotes the Shannon entropy of the moved image M. The Shannon entropy is defined as:

2 $$\begin{aligned} H(T) = -\sum _{t \in T } p_{T}(t) log p_{T}(t), \end{aligned}$$

with $p_{T}(t)$ representing the probability for a voxel in image T to have an intensity of t. The joint entropy H(T, M) of the template image T and the moved image M is calculated by:

3 $$\begin{aligned} H(T, M) = -\sum _{t \in T }\sum _{m \in M }p_{T,M}(t,m) log p_{T,M}(t,m), \end{aligned}$$

where the probability distribution function $p_{T,M}(t,m)$ is obtained from a normalised 2D histogram representing the distribution of every grey value correspondence between the images T and M.

For each group of input volumes, the template image T is calculated anew by using the mean intensity of the input volumes. For pairwise registration, the Mutual Information is calculated between the fixed and moved image.

The Gradient $L_2$ loss is calculated for groupwise and pairwise registration using the deformation fields $\phi$:

4 $$\begin{aligned} L_{2}(\phi ) = \sum _{p\epsilon \Omega } ||\nabla u(p)||^2 , \end{aligned}$$

where p respresents a voxel in the deformation field. Gradient $L_2$ loss is utilised to regularise and smoothen the deformation field.

The Mutual Information and Gradient $L_2$ loss are combined using a $\lambda$ parameter, which acts as a balancing weight for the Mutual Information and Gradient $L_2$ loss. For groupwise registration, the Gradient $L_2$ loss of the n deformation fields is weighted with $\lambda$. The combined loss function is therefore calculated by:

5 $$\begin{aligned} L(T, M, \phi ) = \sum _{i=1}^n L_{MI}(T,M_{i})+\lambda L_{2}(\phi _{i}) \end{aligned}$$

We used five-fold cross-validation. The networks were trained for 200 epochs with early stopping, where training was ended if the validation loss has not improved in five consecutive epochs. We chose five epochs as so-called patience, as we observed that longer patience does not lead to significant improvements while increasing the training time. The model weights with the highest validation accuracy were selected and saved. We used the Adam optimiser and a batch size of 1. We tuned the learning rate and the $\lambda$-parameter by a grid hyperparameter search. The parameter configuration that yielded the highest Mutual Information on the validation dataset was used to train the networks. We resized the network input volumes to 192 x 192 x 64 voxels with a spatial resolution of 2 x 2 x 2 mm^3^ and normalised the intensities of the input volumes to the range [0, 1].

Our experiments were performed using Python with the Tensorflow framework^27^. The networks were trained on a NVIDIA A100 GPU with 80 GB of GPU memory from the bwForCluster Helix (https://wiki.bwhpc.de/e/Helix). Our source code is available at https://github.com/Computer-Assisted-Clinical-Medicine/Groupwise_Multiresolution_Deformable_Medical_Image_Registration.

### Baseline methods

As baseline, we used the classical iterative registration method SimpleElastix. We applied the pairwise bspline algorithm with default parameter settings. The volume of time step 1 is used as fixed image, the remaining volumes are used as moving images. As a second and third baseline method, we used the groupwise registration with SimpleElastix with group sizes 5 and 10 and default parameter settings. To achieve medically plausible registration results, we adjusted the parameter “FinalGridSpacingInPhysicalUnits” to an isotropic 50 mm for SimpleElastix pairwise and groupwise registration.

### Evaluation metrics

We evaluated the registration results using several metrics. We calculated the Structural Similarity Index (SSIM) between the moved images and the template images of the groupwise registration with a group size of 5 three-dimensional volumes (called SSIM 5) and a group size of 10 three-dimensional volumes (called SSIM 10). Additionally, we analysed the degree of image folding as in Strittmatter et al.^21^. Image folding leads to medically implausible deformations and is caused by a Jacobian determinant $\le$ 0 in the deformation field. We calculated the relative value of Jacobian determinants $\le$ 0 in the deformation field. Furthermore, we measured the time required to register a whole four-dimensional DCE-MR scan with 50 three-dimensional volumes / time steps.

**Fig. 2:**
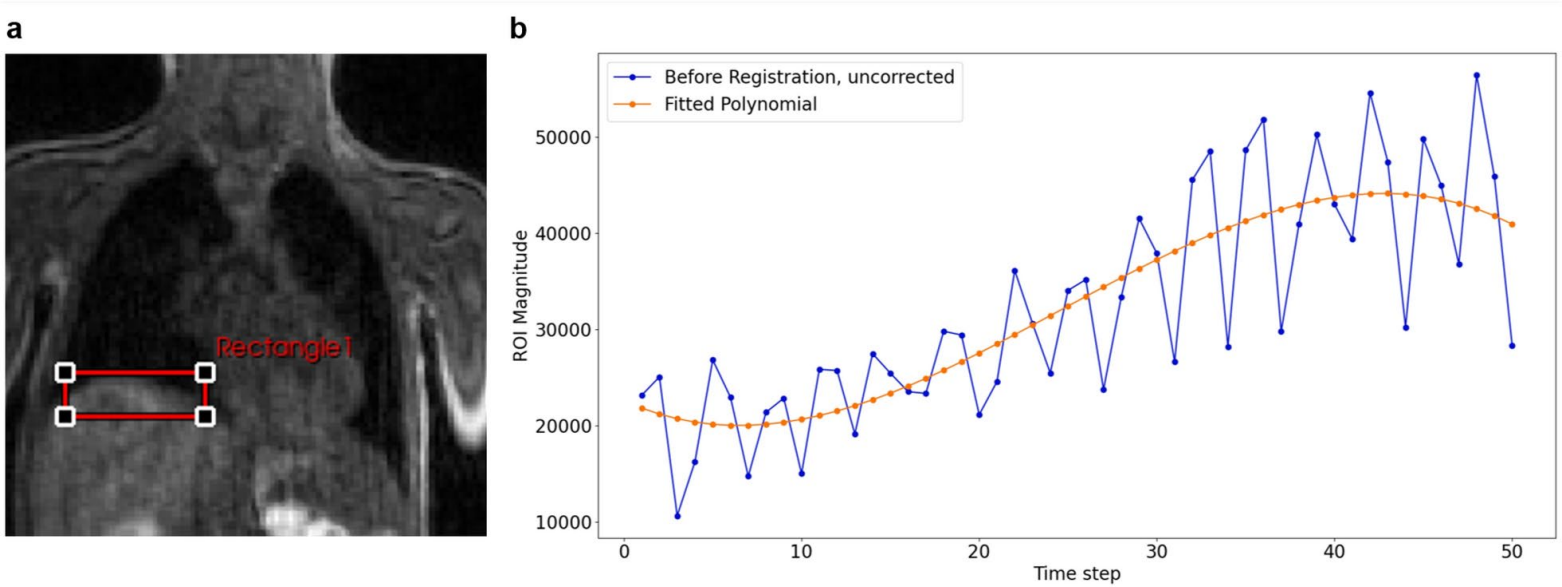
(**a**) ROI position. (**b**) Uncorrected ROI magnitude of the time series before registration plotted in blue and fitted polynomial plotted in orange.

To analyse the deformation of the organs over time / time steps we placed a rectangular region of interest (ROI) on the liver dome (Fig. 2a). We calculated the ROI magnitude, i.e. the sum of the intensity values within the rectangular ROI, and its standard deviation for each time step. The ROI magnitude is dimensionless. The offset of the ROI magnitude was excluded, as this only depends on the intensity of the tissue and contrast agent administration and spread in the body and does not reflect the deformation of the target structures. For this purpose, a second-order polynomial was fitted to the uncorrected ROI magnitude curve (Fig. 2b). This polynomial was subtracted from the ROI magnitude. The corrected ROI magnitude therefore only contains components that are dependent on the deformation of the target structures.

Additionally, we assessed the registration results visually. For this purpose, we displayed the scans of two time steps of a DCE-MR scan. A central coronal slice was selected from the three-dimensional volumes, cropped and overlaid with the lung segmentation. In addition, we colour-coded and overlaid the scans of the two time steps in a composite image. For this, the scan of the first time step was displayed in blue and the scan of the other time step in red. Moreover, the deformation field was analysed visually to identify the regions with larger deformations. For the three-dimensional volumes of individual time steps, the registration methods calculate four-dimensional deformation fields. The last dimension has the value 3, i.e. the displacement is calculated in each of the three spatial directions of the three-dimensional volumes. The deformation field is displayed in RGB colours. The red channel describes the displacement on the y-axis, i.e. the volume’s height, green the displacement on the x-axis/width and blue the displacement on the z-axis/slice direction.

Moreover, for this four-dimensional DCE-MR scan, we calculated the perfusion parameters using the OsiriX plugin UMMPerfusion^28,29^. UMMPerfusion calculates voxel-wise maps of the pulmonary perfusion (PBF), pulmonary blood volume (PBV), and the mean transit time (MTT). For the calculation an arterial input function (AIF) has been carefully selected by placing a ROI in the right pulmonary artery in the unregistered scans for each patient. The ROI was then transferred to all registeration results. In case that the ROI of the AIF did not match the lumen of the pulmonary artery any more (due to the registration), it was replaced to avoid partial volume effects.

As in Weis et al.^30^, to analyse the parametric maps, we then manually positioned three circular ROIs on each lung (ipsilateral and contralateral) apically, centrally and basally. These ROIs were placed through five neighbouring slices to obtain cylindrical ROIs. The ROIs were positioned ventrally, on central slices and dorsally. The mean of the ROIs and the mean standard deviation within the ROIs were calculated to determine whether registration of the DCE-MR scans subsequently resulted in more homogeneous perfusion maps, i.e. a reduction in the standard deviation of the ROIs.

We performed statistical hypothesis tests to assess the statistical significance of the improvements in the SSIM and standard deviation of the ROIs in the perfusion maps. We compared the unregistered data with the registered data of all methods, and the results of the registered methods with each other. We conducted a paired t-test for normally distributed data and a Wilcoxon signed-rank test for non-normally distributed data. Our null hypothesis states that image registration did not lead to improvements in the SSIM and standard deviation of the ROIs in the perfusion maps. Our null hypothesis is rejected if $p < 0.05$. We performed Bonferroni correction.

### Experiment

We evaluated the performance of the proposed groupwise registration network on the four-dimensional CDH dataset using two group sizes. We used groups of 5, called Groupwise 5 network in the following, and groups of 10 three-dimensional volumes / time steps, called Groupwise 10 network in the following. The Groupwise 5 network was trained with a learning rate of 1e-4 and a $\lambda$-parameter of 2. For training the Groupwise 10 network, we chose a learning rate of 1e-4 and a $\lambda$-parameter of 1. The benchmark pairwise network was trained with a learning rate of 5e-4 and a $\lambda$-parameter of 20. As additional benchmark, the GroupRegNet was applied with a group size of 5 and 10, called GroupRegNet 5 and GroupRegNet 10. The GroupRegNet 5 was trained with a learning rate of 1e-5 and a $\lambda$-parameter of 5. The GroupRegNet 10 was trained with a learning rate of 1e-4 and a $\lambda$-parameter of 2.

## Results

Table 1 shows the registration results. The pairwise and groupwise registration with SimpleElastix (groups of 5 and 10) reduced the SSIM 5 and SSIM 10 significantly ($p < 0.00001$) compared to before registration. The proposed groupwise networks were able to increase the SSIM 5 and SSIM 10 significantly ($p < 0.00001$). The Groupwise 5 network produced a significantly higher SSIM 5 ($p < 0.00001$) but significantly lower SSIM 10 ($p < 0.00001$) than the Groupwise 10 network. The pairwise network yielded a similar SSIM 5 ($p = 0.24635$) as before registration but a significantly higher SSIM 10 ($p < 0.00001$) compared to before registration. The SSIM 5 and SSIM 10 of the GroupRegNet 5 network are significantly higher than before registration ($p < 0.00001$), the SSIM 5 is significantly lower ($p < 0.00001$) than the Groupwise 5 network but the SSIM 10 is significantly higher ($p < 0.00001$) than the Groupwise 5 network. Registration with the GroupRegNet 10 network significantly reduced the SSIM 5 ($p < 0.00001$) but significantly ($p < 0.00001$) improved the SSIM 10.

Registration with all neural networks (except GroupRegNet 5 on SSIM 10) resulted in a reduction in the standard deviation of the SSIM 5 and SSIM 10.

All registration methods yielded registration results with a very low degree of image folding ($\vert$J$\vert$ $\le 0$: 0.0 ± 0.0 $\%$). The Baseline SimpleElastix pairwise required more than 13 minutes, SimpleElastix groupwise 5 about 14 minutes and SimpleElastix groupwise 10 about 16 minutes to register a four-dimensional DCE-MR scan (50 three-dimensional volumes / time steps), significantly longer than the neural networks, which required less than 10 seconds.

**Table 1:** The Structural Similarity Index (SSIM), registration time and standard deviation of the corrected liver dome ROI magnitude (mean ± SD) results of the CDH dataset of the baselines SimpleElastix, groupwise networks and the benchmark networks. SSIM: higher value is better (maximum is 1), SD ROI: lower value is better (minimum is 0).

Method/Network	SSIM 5	SSIM 10	Registration Time (s)	SD ROI liver dome
Before registration	0.935 ± 0.104	0.911 ± 0.032	–	2485 ± 1112
Baseline SimpleElastix pairwise	0.927 ± 0.036	0.909 ± 0.039	804.07 ± 153	1628 ± 590
Baseline SimpleElastix groupwise 5	0.894 ± 0.094	0.873 ± 0.093	892.42 ± 85.28	1723 ± 703
Baseline SimpleElastix groupwise 10	0.841 ± 0.147	0.824 ± 0.143	977.53 ± 76.98	1783 ± 684
Groupwise 5	0.953 ± 0.025	0.935 ± 0.031	8.55 ± 0.51	1516 ± 665
Groupwise 10	0.951 ± 0.021	0.936 ± 0.028	9.07 ± 0.56	1018 ± 1660
Benchmark pairwise	0.937 ± 0.021	0.924 ± 0.027	9.81 ± 0.60	2037 ± 801
Benchmark GroupRegNet 5	0.951 ± 0.023	0.942 ± 0.036	2.26 ± 0.17	1728 ± 775
Benchmark GroupRegNet 10	0.934 ± 0.022	0.930 ± 0.027	3.14 ± 0.06	2377 ± 1141

Table 1 column “SD ROI liver dome” shows the standard deviation of the corrected liver dome ROI magnitudes over the time steps of the whole CDH dataset before registration and after registration with the different registration methods. All registration methods except the benchmark pairwise network were able to significantly reduce the standard deviation of the corrected liver dome ROI magnitude ($p < 0.00001$). The Groupwise 10 network yielded the lowest standard deviation of the corrected liver dome ROI magnitude.

**Fig. 3:**
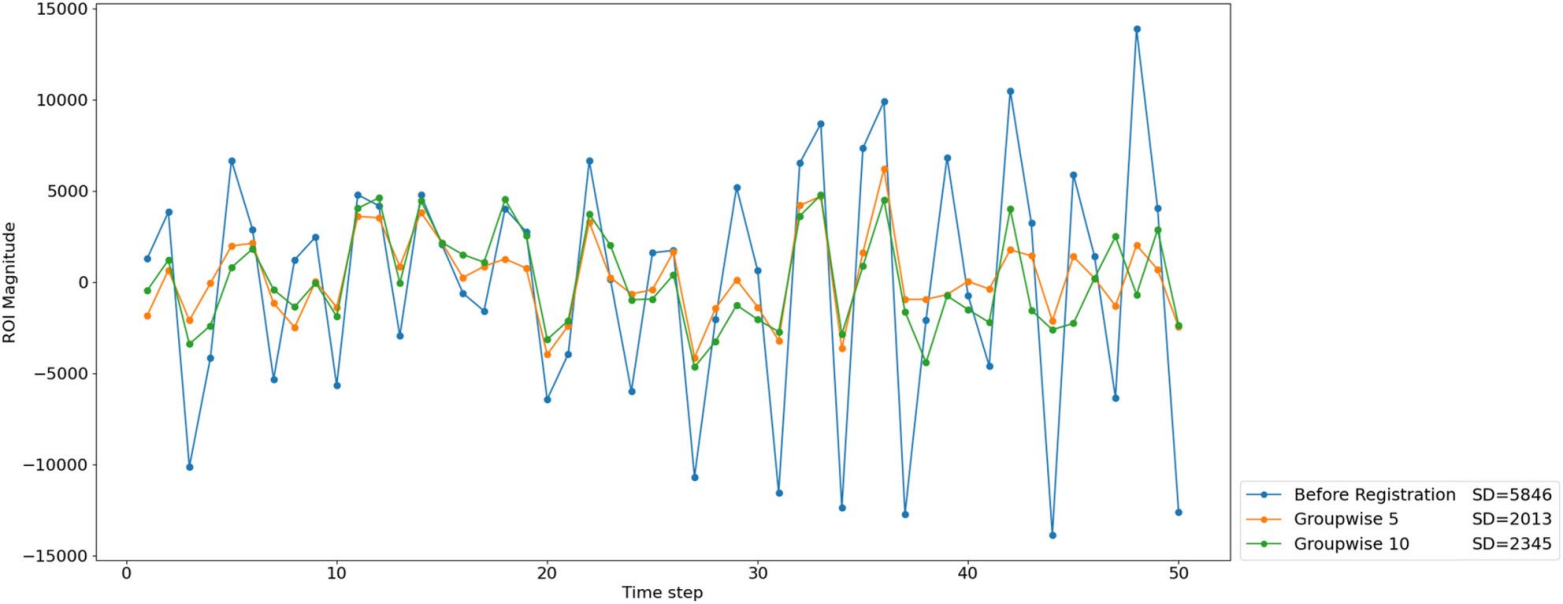
An example of a corrected liver dome ROI magnitude (arbitrary units) of one patient’s four-dimensional DCE-MR scan over the time steps before and after registration with the Groupwise 5 and Groupwise 10 networks. The standard deviation (SD) of each signal for this one patient is displayed.

Figure 3 shows examples of the corrected liver dome ROI magnitude of one patient before and after registration plotted over the timesteps. To ensure clarity, we do not show the ROI magnitude of all methods in this graph, instead we display the ROI magnitude before registration and after registration with our Groupwise 5 and Groupwise 10 networks. The curve before registration contains large breathing artefacts due to non-uniform deformation of the target structures in the ROI across the time steps. The ROI magnitude curve is smoothed by registration, i.e. by spatial alignment of the corresponding structures in the volumes of the different time steps. The best results, i.e. curve with the lowest standard deviation, was achieved with the Groupwise 5 network.

**Fig. 4:**
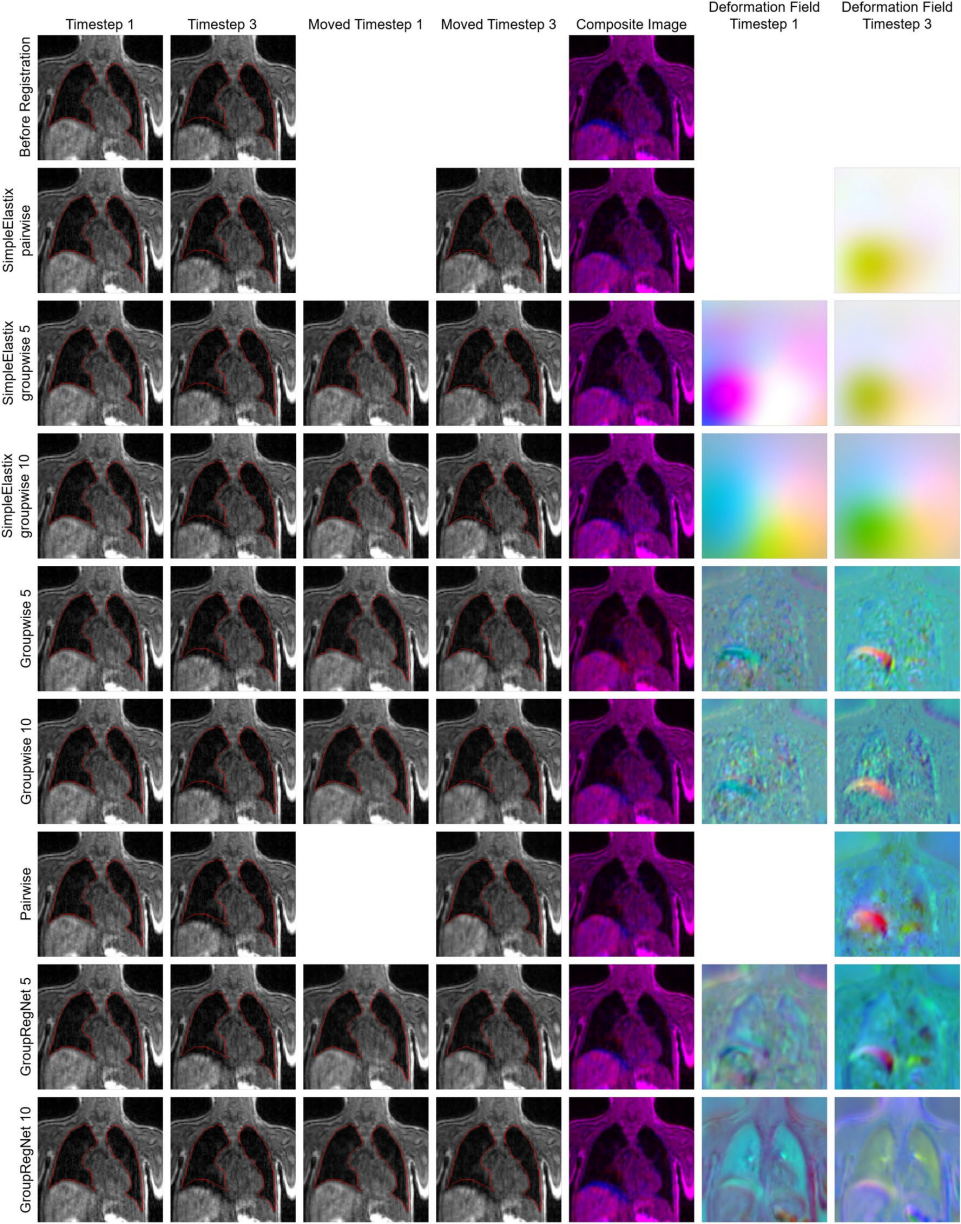
Example results of the registration of two volumes/time steps from the CDH dataset. The cropped images show central slices of the coronal plane. The MR scans are overlaid by the lung segmentations (column 1–4). The resulting composites show the data of the first time step in blue and the data of the third time step in red. The deformation field is visualized in RGB channels.

Figure 4 shows examples of registration results. Here, we chose the same DCE-MR scan for which we also plotted the ROI magnitude (Fig. 3). In Fig. 4, before registration, especially the liver dome and smaller lung structures are not spatially aligned well. Registration with all registration methods except GroupRegNet10 yielded a high overlap of the corresponding structures. The deformation fields of the Baselines SimpleElastix pairwise and groupwise are smooth, the largest deformations are located in the area of the right abdomen. The deformation fields of the neural networks indicate that the largest deformation of corresponding structures between the different time steps occurred at the liver dome. Additionally, the spatial alignment was corrected for finer lung structures.

The perfusion maps (Table 2) were calculated for the DCE-MR scan for which we also plotted the ROI magnitude and evaluated the registration results visually in Fig. 4. Our proposed groupwise networks (Groupwise 5 and Groupwise 10) significantly reduced the standard deviation of the ROIs on the maps of the mean transit time, pulmonary blood volume and pulmonary blood flow. All registration networks significantly reduced the standard deviation of the ROIs on the maps of the pulmonary blood volume and pulmonary blood flow. For the registration results of the baseline SimpleElastix groupwise 5, the pulmonary blood volume and pulmonary blood flow were incorrectly overestimated.

**Table 2:** Perfusion maps: mean transit time (MTT), pulmonary blood volume (PBV) and pulmonary blood flow (PBF) (mean ± SD) calculated on the volumes before registration and the results of the baselines SimpleElastix, groupwise networks and the benchmark networks. With the bold numbers indicating significant improvement (p-value < 0.05) to the unregistered data (first row).

Method/network	MTT (s)	PBV (ml/100ml)	PBF (ml/100ml/min)
Before registration	5.72 ± 1.96	6.36 ± 2.74	70.16 ± 26.28
Baseline SimpleElastix pairwise	5.67 ± 2.08	5.59 ± **2.24**	64.41 ± **23.67**
Baseline SimpleElastix groupwise 5	6.16 ± 2.19	9.46 ± 3.31	104.49 ± 32.19
Baseline SimpleElastix groupwise 10	5.22 ± 1.75	7.76 ± 3.26	88.97 ± 28.04
Groupwise 5	5.71 ± **1.73**	6.35 ± **2.43**	70.76 ± **21.78**
Groupwise 10	6.40 ± **1.62**	6.76 ± **2.38**	67.99 ± **20.07**
Benchmark pairwise	5.91 ± 1.73	6.60 ± **2.17**	72.22 ± **22.20**
Benchmark GroupRegNet 5	5.94 ± 1.80	6.04 ± **2.24**	65.65 ± **22.10**
Benchmark GroupRegNet 10	5.91 ± 1.82	6.12 ± **2.30**	67.79 ± **23.77**

Furthermore, we monitored the RAM and VRAM usage during training of the Groupwise networks. The Groupwise 5 network used a maximum of 12.8 GB RAM and 35.1 GB VRAM during training, whereas the Groupwise 10 network used a maximum of 25.1 GB RAM and 69.7 GB VRAM during training.

## Discussion

We proposed groupwise multiresolution deep learning networks for the deformable registration of DCE-MR time series for the assessment of lung perfusion in patients with CDH. By comparing the groupwise networks with a pairwise registration network as benchmark and a pairwise registration with SimpleElastix as baseline, we investigated the impact of using groupwise vs pairwise registration on the registration result. Additionally, we compared the proposed groupwise registration network to an iterative groupwise registration method SimpleElastix and a published groupwise registration network to show the superiority of the proposed groupwise registration network. For our proposed groupwise network, we chose a network architecture that uses multiple resolutions, thus combining the advantages of groupwise and multiresolution registration.

Recently published DCE-MRI image registration methods address the challenge of multimodal image registration. For instance, Cai et al.^8^ apply an image-to-image translation network to convert the multimodal image registration into a monomodal image registration task. Sun et al.^10^ also focus on reducing the intensity changes induced by contrast agent administration and, additionally, applies a coarse-to-fine image registration approach in which pairwise registration is used to compute a coarse transform and groupwise registration is used to refine the transformation. In our study, we focused on using groupwise multiresolution registration. In order to address the challenge of multimodal image registration, we used the entropy-based loss function Mutual Information to train the networks as well as a basic network architecture that has already demonstrated high performance on four multimodal medical datasets^21^.

In recent years, several groupwise medical image registration methods have been published, these include iterative/classical methods^14,31–36^ and deep learning-based approaches^22,37–44^. Only one published neural network for groupwise registration also applies a multiresolution approach^43^, and one network uses a coarse and a fine network to first reduce larger image displacements and then correct finer deformations^40^. As a benchmark network, we used the published groupwise registration network GroupRegNet^22^, as it was developed and evaluated on two publicly available four-dimensional CT thorax datasets and outperformed several published medical image registration methods.

Assessing the networks’ performance is challenging due to the lack of ground truth data^6^. To address this problem, we applied different methods to evaluate the registration results. We used the SSIM to compare the structural similarity between the volumes, focusing on luminance, contrast and structure. We analyzed the deformation field by calculating the Jacobian determinant at each point to detect any image folding. Furthermore, we calculated the standard deviation of the liver dome ROI magnitude to track changes in the deformation of the liver dome over the time steps. Additionally, a visual inspection of the deformation field was performed to identify areas with major deformation and to check whether the deformation was smooth within the individual organs. Moreover, we analyzed the homogeneity of the perfusion maps mean transit time, pulmonary blood volume and pulmonary blood flow.

Pairwise and groupwise registration with SimpleElastix resulted in a significant reduction of the SSIM. In some three-dimensional volumes, larger circular areas with uniform intensity (standard pixel value of the registration algorithm) appear in the first and last two slices due to the large grid spacing of the registration methods. Therefore, in medical image analysis, the perfusion cannot be evaluated in these slices. For SimpleElastix groupwise 5, the ROI placement for the UMMPerfusion algorithm was challenging, therefore affecting the perfusion calculation.

We evaluated the groupwise registration network using two group sizes. In our proposed network, using groups of five volumes performed better than groups of ten volumes in SSIM 5, but worse in SSIM 10. The standard deviation of the liver dome ROI magnitude and the standard deviation of the ROIs of the perfusion maps are significantly lower after registration with the Groupwise 10 network than after registration with the Groupwise 5 network. The visual evaluation demonstrated that the spatial alignment was considerably improved for both group sizes compared to before registration.

The proposed groupwise networks achieved significantly better results than the pairwise network, based on SSIM and standard deviation of liver dome ROI magnitude, and resulted in a low degree of image folding (number of $\vert$J$\vert$ $\le 0$). For the standard deviation of the ROIs of the perfusion maps the results were mixed. For the pairwise network, a comparatively high lambda parameter had to be set in order to achieve medically plausible registration results for the scans of all time steps. With less regularization of the displacement field, good registration results could still be achieved for the first 5-10 timesteps. In subsequent time steps, corresponding structures in the fixed and moving image differed in intensity due to the contrast agent administration, which made registration difficult and led to medically implausible deformation of the liver and kidneys. Here we had to compromise between a high deformation correction, that is, improved spatial alignment through registration (low lambda-parameter) and a smooth deformation field that leads to medically plausible transformations (high lambda-parameter).

The proposed Groupwise network outperformed the benchmark GroupRegNet network (Groupwise 5 vs GroupRegNet 5 and Groupwise 10 vs GroupRegNet 10, except for SSIM 10 and the standard deviation of the ROIs of the pulmonary blood volume map). The reason for this could be that the monoresolution approach of the GroupRegNet is not able to correct the varying degrees of deformation, especially in the liver and lungs. Whereas the proposed groupwise network utilises multiple resolutions to correct initially coarser and then increasingly finer deformations.

Different from the deformation fields generated by neural networks, the deformation fields generated by the baselines SimpleElastix pairwise and groupwise did not show any recognizable structures such as organ contours. This is due to the high value of the final grid spacing parameter (“FinalGridSpacingInPhysicalUnits”) of 50 x 50 x 50 mm^3^, which ensures smooth and medically plausible transformations. Using a smaller grid spacing led to unrealistic deformations. For the neural networks, the grid spacing was set to 2 x 2 x 2 mm^3^, with the degree of deformation being controlled by the lambda parameter. For groupwise registration with SimpleElastix, no regularisation loss can be added as of now, so the smoothness of the deformation field cannot be controlled via a lambda parameter.

The registration process with the baseline took significantly longer than with the neural networks. This difference results from SimpleElastix using an iterative algorithm in contrast to deep learning-based methods. While GPU implementations^45^ or techniques that involve local similarity estimations^46^, can drastically reduce the time required for registration, SimpleElastix was chosen for this study due to its wide acceptance and use in a wide variety of medical image registration applications. The registration with the proposed groupwise networks took significantly longer than with the benchmark GroupRegNet, as in the proposed groupwise network several networks were cascaded for a multiresolution approach, whereas in the benchmark GroupRegNet a single U-Net^47^ with only one resolution was used.

In our study, the network training was limited by the available GPU memory (NVIDIA A100 GPU with 80 GB), limiting the group size for the groupwise registration networks to 10. Nevertheless, we were able to demonstrate the effectiveness of groupwise registration networks for DCE-MR image registration.

## Conclusion

We developed neural networks for groupwise deformable registration of medical images, especially for DCE-MRI image registration. Our experiments demonstrate that registration with the groupwise networks results in high spatial alignment, medically plausible transformations, and fast registration of less than 10 seconds for a DCE-MR scan. Additionally, our results show that image registration with our proposed groupwise network results in more homogeneous perfusion maps, thereby improving the accuracy of medical image analysis.

## Electronic supplementary material

Below is the link to the electronic supplementary material.

Supplementary Information.

## Data Availability

Our source code is available at https://github.com/Computer-Assisted-Clinical-Medicine/Groupwise_Multiresolution_Deformable_Medical_Image_Registration. The dataset analysed during this study is not publicly available due to data protection and patient privacy regulations but is available from the corresponding author on reasonable request.
