# Supplementary material for: A groupwise multiresolution network for DCE-MRI image registration
Source: Sci Rep. 2025 Mar 22;15:9891. doi: 10.1038/s41598-025-94275-9 (PMC11929895; doi:10.1038/s41598-025-94275-9)
Supplement: Supplementary file 1 — Supplementary Information. [file 41598_2025_94275_MOESM1_ESM.pdf]

A Groupwise Multiresolution Network for DCE-MRI Image Registration

Anika Strittmatter, Meike Weis, and Frank G. Zöllner

Pairwise and Groupwise Network Training

Image registration methods can be divided into pairwise or groupwise methods. In pairwise registration, two images are registered where one image is defined as the reference/fixed image and the other image is the moving image. During registration, a transformation is calculated which is then applied to the moving image, resulting in the moved image. The network inputs are the fixed image and the moving image. In our experiments, the pairwise networks were trained unsupervised according to the VoxelMorph framework (Figure 1). During training, the image similarity between the fixed and the moved image is analyzed using Mutual Information. During training, the Adam Optimiser adjusts the network weights in order to improve the image similarity, i.e. the spatial alignment of the fixed and moved image, while maintaining a smooth transformation (Gradient L2 loss).

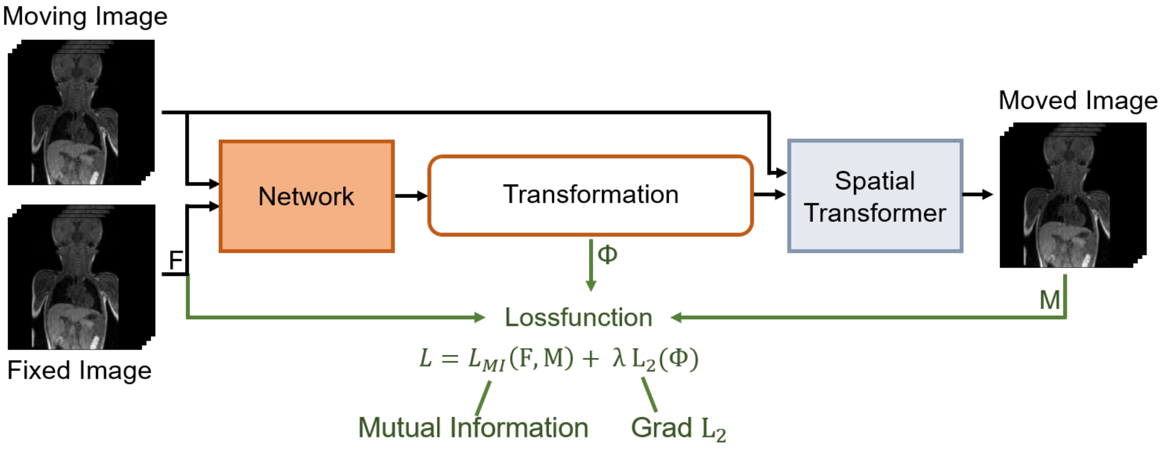

Figure 1. Unsupervised training of the pairwise networks, based on the VoxelMorph framework.

In Groupwise image registration setting a fixed image is usually omitted. Instead, all input images are moving images. During registration, a transformation is calculated for all input images and is applied to the moving images. Thus, after registration, n moved images are created from n input images. The average intensity image is calculated from all n input images to form a template image. In our experiments, all groupwise networks were trained unsupervised (Figure 2). During training, the image similarity of the resulting n moved images was compared with the template image using Mutual information, additionally Gradient L2 was calculated for every transformation.

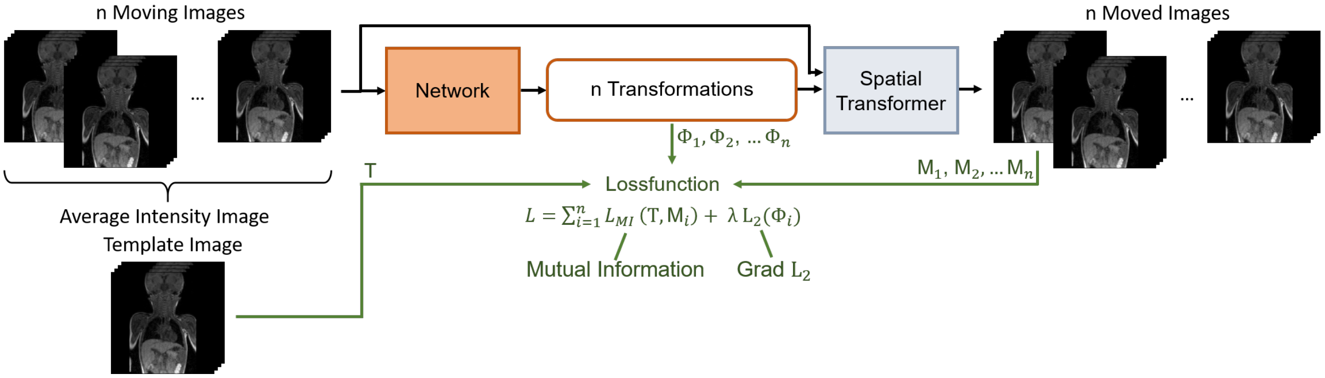

Figure 2. Unsupervised training of the groupwise networks.

Perfusion Maps

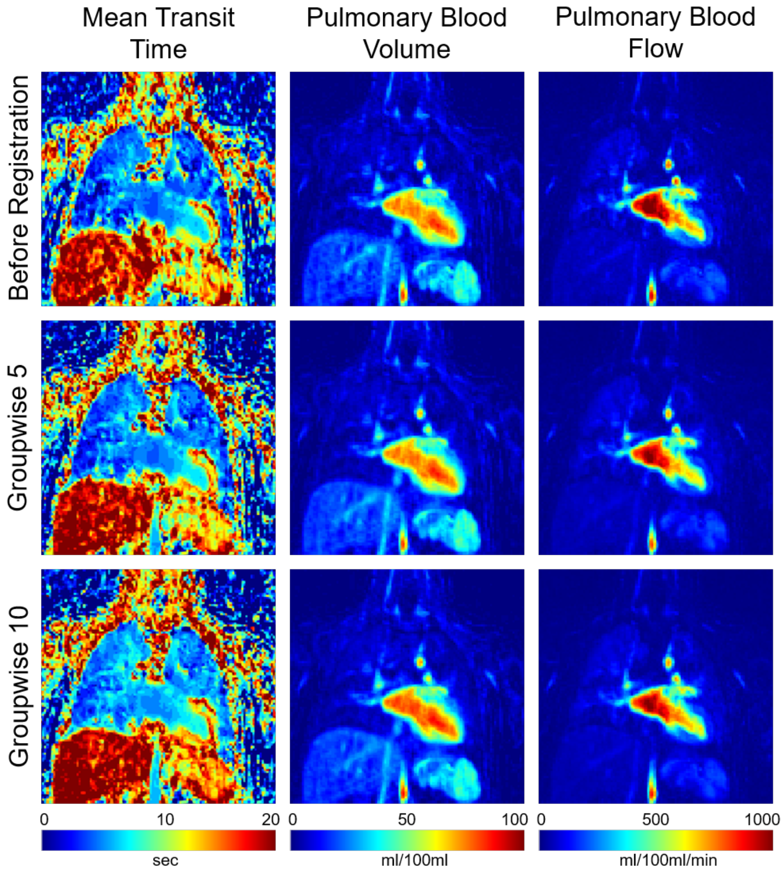

Figure 3. Example of perfusion maps generated by UMMPerfusion of one representative patient. From left to right mean transit time (sec), pulmonary blood volume (ml/100ml), and pulmonary blood flow (ml/100ml/min) are shown. Top row represents before registration, second and third row represents results from our proposed groupwise deep learning network.
